# Familial Prenatal Total Anomalous Pulmonary Venous Drainage: Genetic Implications and Multimodal Echocardiographic and MRI Assessment

**DOI:** 10.1016/j.jaccas.2025.104551

**Published:** 2025-08-06

**Authors:** Giovanni Granozio, Mari Nieves Velasco Forte, Alexia Egloff, Tomas Woodgate, Karen J. Low, Ayse Nur Kavasoglu, Hannah Bellsham-Revell, Kuberan Pushparajah, Laura Vazquez-Garcia, David F.A. Lloyd

**Affiliations:** a Department of Congenital Heart Disease, Guy's and St Thomas' NHS Foundation Trust, London, United Kingdom; b Department of Congenital Heart Disease, University Hospital Bristol, Bristol, United Kingdom; c Department of Radiology, Guy's and St Thomas' NHS Foundation Trust, London, United Kingdom; d School of Biomedical Engineering and Imaging Sciences, King's College London, London, United Kingdom; e Department of Clinical Genetics, University Hospitals Bristol and Weston NHS Trust, Bristol, United Kingdom; f Centre for Academic Child Health, Bristol Medical School, University of Bristol, Bristol, United Kingdom

**Keywords:** cardiac magnetic resonance, congenital heart defect, echocardiography, three-dimensional imaging, ultrasonography

## Abstract

We report 2 cases of fetal total anomalous pulmonary venous drainage (TAPVD) diagnosed in subsequent pregnancies in the same patient. In the first pregnancy, supracardiac TAPVD with obstruction at the ascending vein was identified at 20 weeks. Three-dimensional (3D) motion-corrected fetal cardiac magnetic resonance imaging (MRI) aided visualization of the venous pathway and revealed subtle T2-weighted lung heterogeneity, suggesting secondary pulmonary lymphangiectasia. The baby was delivered in cardiac theatres, with surgical repair at 4 hours of life. In a subsequent pregnancy, infracardiac TAPVD was diagnosed at 15 weeks using 3D fetal echocardiography. No pulmonary venous obstruction was present. Third-trimester fetal cardiac MRI confirmed normal lung appearances. Neonatal surgical repair again occurred at 4 hours of age. Genetic testing, including microarray comparative genomic hybridization of the index child and trio whole-genome sequencing of both parents and the index child, identified no causative variants. Familial TAPVD, though rare, should prompt careful fetal echocardiographic screening in subsequent pregnancies. Multimodal imaging with echocardiography and MRI is a useful aid to fetal diagnosis and perinatal planning strategies.

## Graphical Abstract

**Figure:**
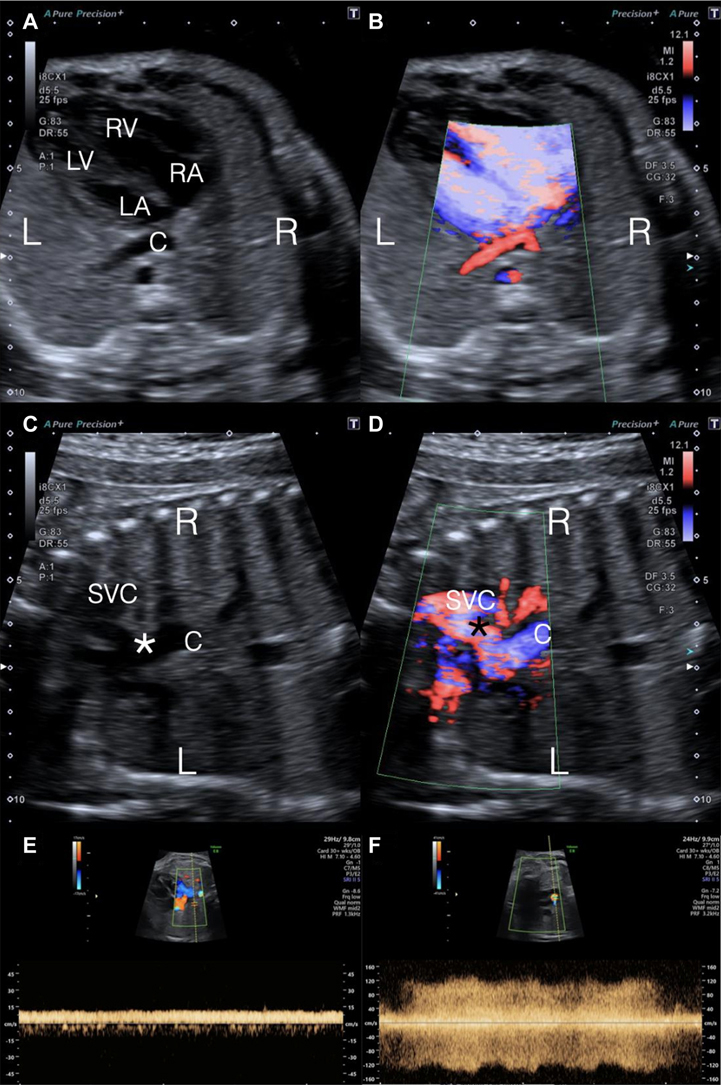

## Case 1

**Figure 1:**
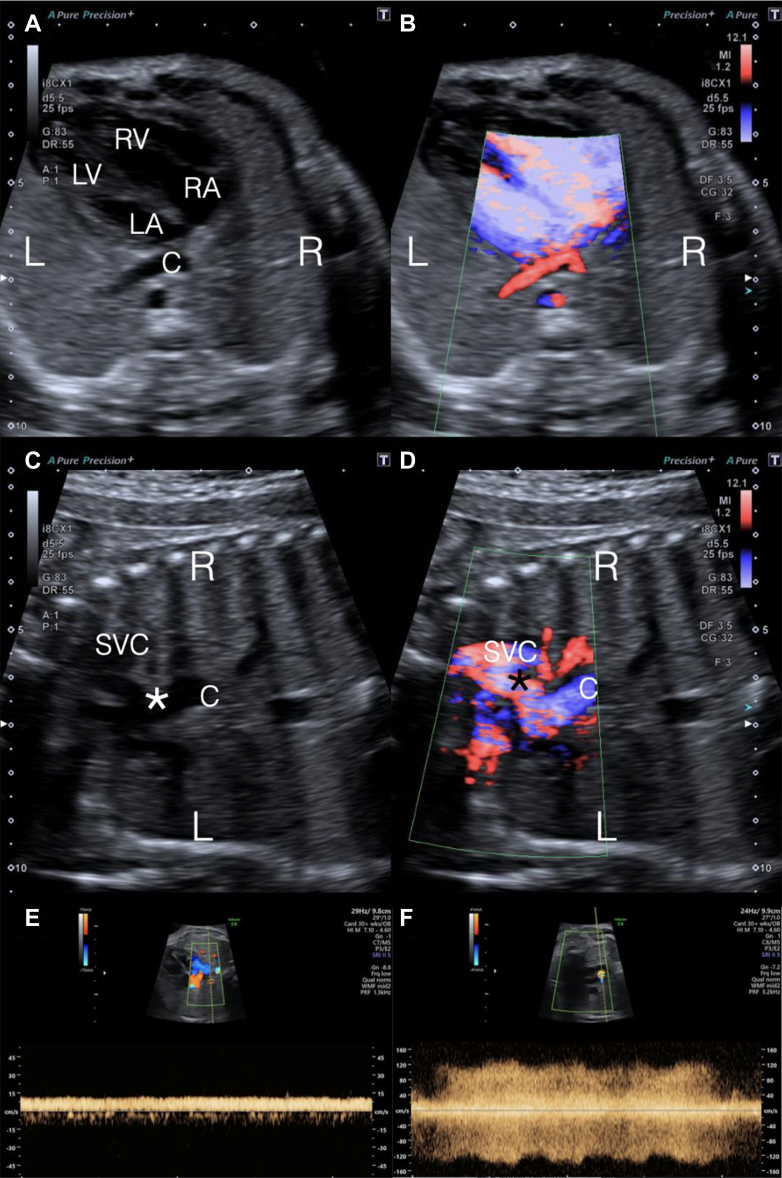
Fetal Echocardiography at 32 Weeks of Gestation (Case 1) (A) Four-chamber view. (B) Four-chamber view with power color Doppler. (C) Coronal view (fetal head to the left). (D) Coronal view with power color Doppler. There is a confluence (C) behind the left atrium (LA) with no direct connection of the pulmonary veins to the cardiac mass. The confluence drains via an ascending vein (asterisk) to the junction of the innominate vein and superior vena cava (SVC). Doppler assessment showed a flat, nonphasic signal in the pulmonary veins (E) with an area over turbulence in the ascending vein (F). L = left; LV = left ventricle; R = right; RA = right atrium; RV = right ventricle.

A 35-year-old primigravida was referred at 20 weeks of gestation from mid-trimester screening. Past medical history and family history were unremarkable with a low-risk early pregnancy screening. Fetal echocardiography demonstrated total anomalous pulmonary venous drainage (TAPVD) with all pulmonary veins draining to a confluence, which decompressed via an ascending vertical vein, passing between the branch pulmonary arteries and joining the posterior aspect of the superior vena cava. Doppler evaluation showed a flat profile in the pulmonary veins with an area of turbulence (maximum velocity 1.4 m/s) in the ascending vein (Figures 1A to 1F).

**Take-Home Messages**

• Recurrent TAPVD is rare but well described, and careful exclusion should be considered in all subsequent pregnancies once an index case is identified.
• Prenatal multimodal imaging with 2D/3D fetal echocardiography and fetal MRI enhances the diagnosis and risk stratification in TAPVD.
• Although genetic variants are rarely found in recurrent isolated TAPVD, genetic counseling remains vital to stay abreast of advances, support reproductive planning, and guide management should a genetic variant be identified.

**Figure 2:**
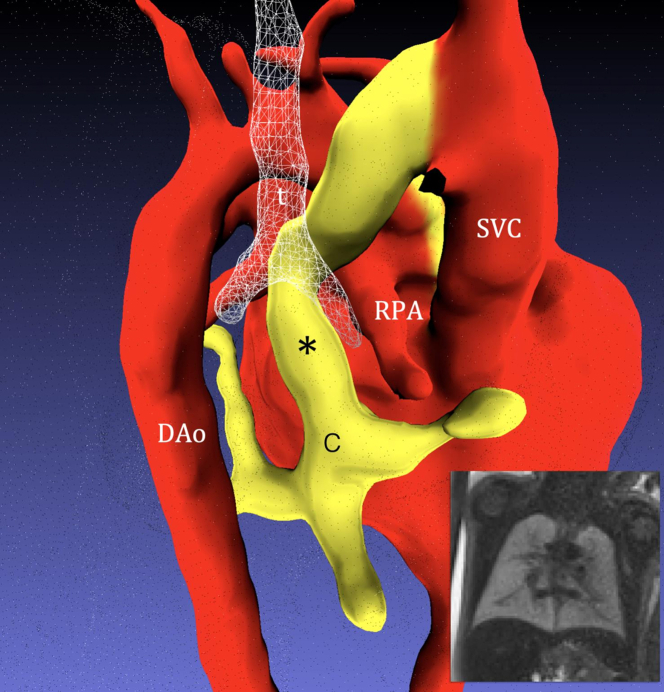
Segmentation From Motion-Corrected Slice-to-Volume Magnetic Resonance Imaging Data (Posterior Rightward Projection) at 34 Weeks The confluence (C) drains via an ascending vein (asterisk) that passes between the right main bronchus (white mesh) and right pulmonary artery (RPA) before draining the innominate vein/superior vena cava (SVC). Inset shows single-shot fast spin echo image of the fetal lungs, demonstrating heterogeneity in the lung parenchyma with small cystic areas suspicious for pulmonary lymphangiectasia. DAo = descending aorta; t = trachea.

Fetal cardiac magnetic resonance imaging (MRI) was performed at 34 + 6 weeks of gestation. Overlapping T2-weighted single-shot fast spin echo sequences (repetition time 20,000 ms, echo time 80 ms [180 ms for lungs], flip angle 90°, voxel size 1.25 × 1.25 mm, slice thickness 2.5 mm) were acquired and post-processed using motion-corrected slice-to-volume registration.^1^ Three-dimensional (3D) visualization of the ascending vein demonstrated a narrowing as it coursed between the right main bronchus and right pulmonary artery. Heterogeneity of the lung parenchyma was noted with small cystic areas, suspicious for pulmonary lymphangiectasia (PL) (Figure 2).

Following multidisciplinary review, arrangements were made for elective cesarean section with the cardiothoracic surgical team on standby in an adjacent operating room. The baby was delivered by elective cesarean section at 38 + 5 weeks of gestation, weighing 3,100 g. He required no immediate support and so was transferred to intensive care under close observation. At 2 hours, he was electively intubated and ventilated owing to evolving respiratory distress and proceeded to surgical repair at 4 hours of age. No pleural effusions were noted at any stage during recovery and he was discharged in stable condition. Balloon dilation of the pulmonary venous confluence was performed at 1 year of age, which was complicated by a right middle cerebral artery stroke leading to left hemiparesis, which has predominantly resolved. At 4 years of age, he is well, asymptomatic, and thriving with no residual obstruction.

## Expert Commentary

Predicting the urgency of surgical intervention in fetal TAPVD can be challenging, with implications to the mode, timing, and location of delivery. In this case, clear prenatal evidence of pulmonary venous obstruction on two-dimensional (2D) and 3D imaging, combined with subtle MRI findings of PL, contributed to the decision to deliver in proximity to cardiac surgical facilities.

## Case 2

**Figure 3:**
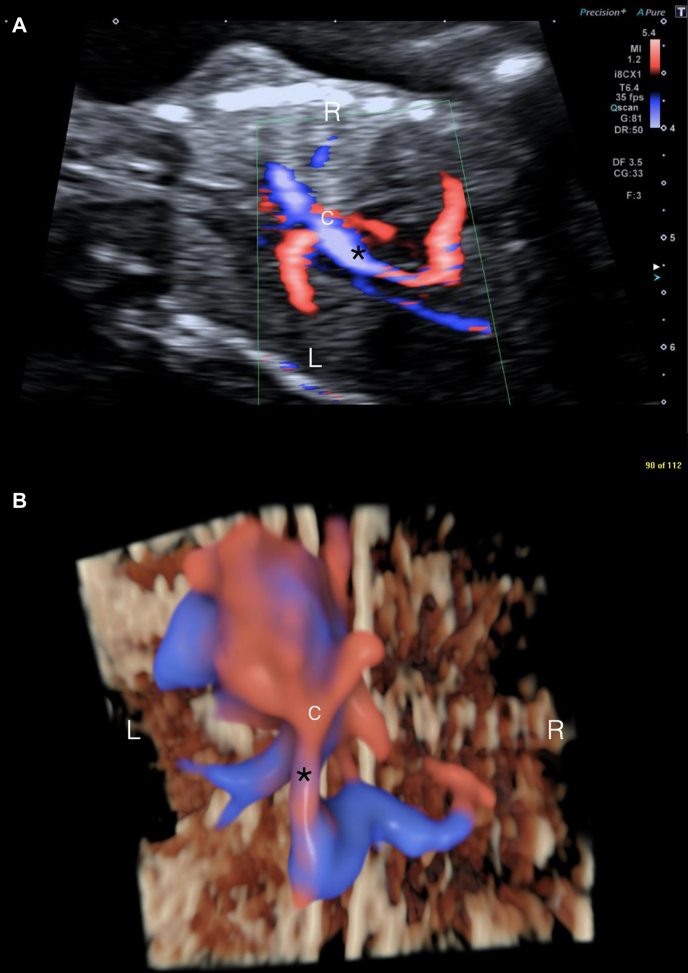
Fetal Echocardiography at 15 Weeks Gestation (Case 2) (A) Coronal view of the fetal chest (fetal head to the left of the image, feet to the right). (B) Three-dimensional color spatiotemporal image correlation image (posterior projection), acquired on a GE Voluson system equipped with an eM6C G3 matrix array probe, at 15 weeks of gestation in a subsequent pregnancy. There is infracardiac total anomalous pulmonary venous drainage with the confluence (C) draining via a descending vein (asterisk) to the portal system. L = left; R = right.

Three years later, the patient was referred again for fetal cardiology review in a subsequent pregnancy at 15 + 6 weeks of gestation. Fetal echocardiography identified a confluence behind the left atrium that decompressed via a descending vein, coursing below the diaphragm to the portal system, demonstrated on 2D and 3D echocardiography (Figures 3A and 3B). Pulmonary venous Doppler studies showed a phasic flow pattern with low-velocity flow in the descending vein.

**Figure 4:**
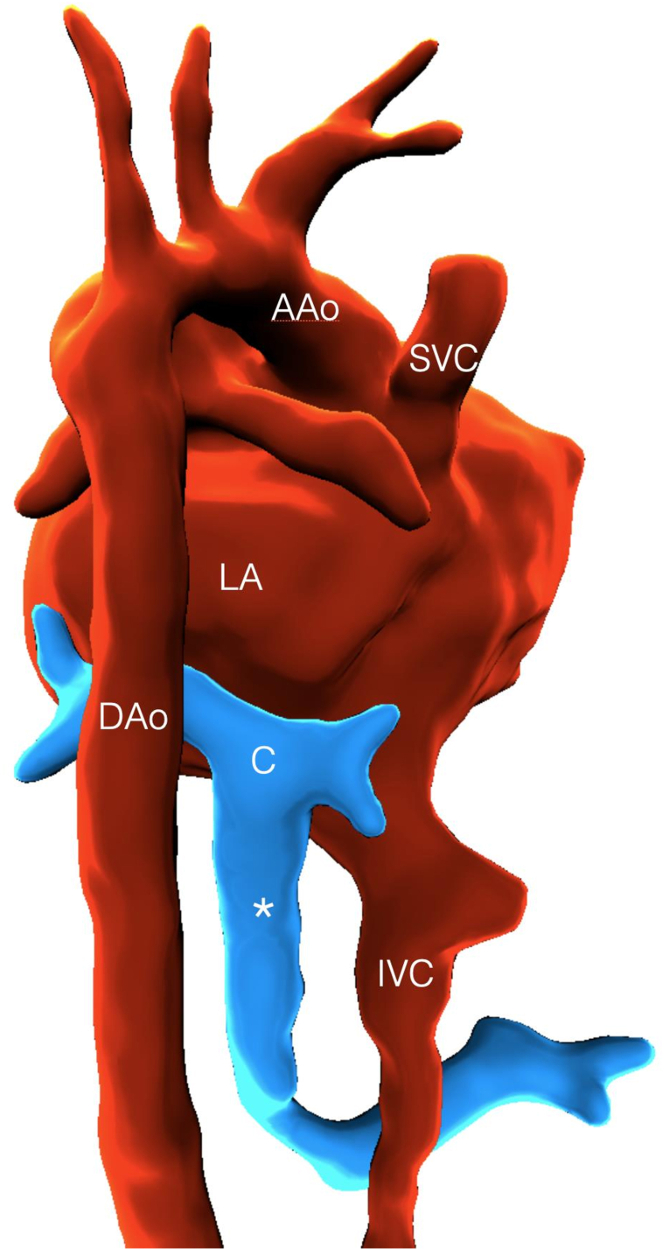
Segmentation From Motion-Corrected Slice to Volume Fetal Magnetic Resonance Imaging Data (Posterior Rightward Projection) in the Subsequent Pregnancy at 32 Weeks (Case 2) Four pulmonary veins drain to a single confluence (C), which drains via a descending vein (asterisk) to the portal system. AAo = ascending aorta; DAo = descending aorta; IVC = inferior vena cava; LA = left atrium; SVC = superior vena cava.

Fetal MRI at 31 + 5 weeks visualized all 4 pulmonary veins draining into the confluence, which decompressed via a descending vertical vein (Figure 4). The fetal lungs were reported as having a normal appearance with no signs of lymphangiectasia.

Clinical genetics consultation did not elucidate any syndromic or additional features; however, given that this was the patient’s second affected pregnancy, rapid whole-genome sequencing (WGS) was performed as a trio with both parents and the index child. No causative gene variants associated with TAPVD were identified. The index child also underwent microarray comparative genomic hybridization (CGH) testing (Oxford Gene Technology 8 × 60K constitutional v3.0), which did not identify any clinically significant copy number variants. Both parents also had normal echocardiograms.

The baby was delivered via elective cesarean section at 39 weeks, with a birth weight of 3,426 g. Postnatal echocardiography confirmed the antenatal diagnosis. Surgical repair of the infracardiac TAPVD was performed on a semielective basis at 4 hours of life owing to signs of intrahepatic and diaphragmatic obstruction on echocardiography. The baby was discharged in good condition and continues well at 1 year of age with no signs of obstruction post-surgery. Postnatal genetic testing also revealed a normal array CGH.

## Expert Commentary

The offer of a detailed fetal echocardiogram in this subsequent pregnancy owing to the previous history was critical in achieving a timely diagnosis here. Although there were no clear signs of pulmonary venous obstruction in this case, infracardiac TAPVD is known to carry a higher risk of early postnatal obstruction compared with supracardiac types, highlighting the challenges in predicting transitional hemodynamics. Most nonsyndromic cases of TAPVD do not have genetic causes, although rare chromosomal copy number changes have been reported. In this context, both index CGH array and trio WGS were considered appropriate following the recurrence in a second child. Whereas no causative variant was identified, clinical genetics input remained valuable in guiding parental counseling and discussions regarding recurrence risk.

## Discussion

Isolated TAPVD accounts for 1% to 5% of all congenital heart disease; however, rates of prenatal detection remain low. Drainage is most commonly supracardiac (to the innominate vein/superior vena cava) in 49% of cases, with infracardiac drainage to the portal system in 26%. Direct cardiac drainage or mixed types account for the remainder. Postnatal obstruction of the pulmonary venous pathway is a surgical emergency, occurring in about 50% of supracardiac TAPVD and 80% to 90% of infracardiac cases.^2^

Whereas fetal diagnosis allows for timely provision of appropriate neonatal care,^3^ predicting the urgency of postnatal intervention can be challenging. Several echocardiographic markers have been proposed to help identify high-risk fetuses, including loss of normal phasic flow patterns in the pulmonary veins and/or increased mean velocity in the ascending vein.^4^^,^^5^ Delineation of the pulmonary venous pathway using 3D imaging with both echocardiography^6^ and MRI^1^ may also be useful. Although not yet widely adopted in routine fetal cardiology practice, our experience demonstrates that these techniques are both feasible and clinically valuable in complex cases. However, in fetal life, the patency of the ductus venosus (which may form part of the drainage pathway in infracardiac TAVPD) and the arterial duct (which may contribute to external compression in supracardiac TAPVD) presents additional challenges to predicting postnatal hemodynamics. In the current case, there were signs of obstruction on both fetal echocardiography and lung MRI in the first case (supracardiac), but there were no prenatal signs of obstruction in the second case (infracardiac); however, early signs of obstruction ultimately developed in both cases requiring surgical repair within hours of delivery.

Secondary PL has been associated with poor outcomes in the context of obstructed TAPVD;^7^^,^^8^ however, clear signs of pulmonary venous obstruction on echocardiography are not universally associated with PL, and vice versa.^7^^,^^9^ In this case, the first fetus demonstrated features suggestive of PL on fetal lung MRI, which, in conjunction with persistent evidence of venous obstruction, informed the decision to plan delivery adjacent to a cardiac operating room. Although no specific postnatal thoracic imaging or lung biopsy was performed, it is notable that the infant did not require immediate postnatal intervention and experienced no specific perioperative respiratory complications, and the child has continued to thrive following surgical repair.

Finally, familial cases of TAPVD are rare but well described: in a cohort of 422 patients with TAPVD, 1.4% had siblings or first-degree relatives with the condition.^3^ TAPVD has been found in conjunction with several genetic syndromes, including mutations in *GATA4* and *ZIC3*, as well as cardiofaciocutaneous syndrome, Holt-Oram syndrome, Williams syndrome, and cat-eye syndrome, suggesting a possible genetic predisposition.^10^ In the current case, no causative variant was identified despite trio WGS testing on the index case and both parents. From an embryologic perspective, the common pulmonary vein arises from the splanchnic plexus and incorporates into the left atrium; failure of common pulmonary vein incorporation may lead to persistent connection with systemic veins via the cardinal vein system (supracardiac) or umbilicovitelline system (infracardiac), depending on the stage of development. The variability in TAPVD subtypes in this case does not therefore in itself exclude an underlying genetic cause, albeit one beyond current testing protocols.

## Conclusions

Multimodality imaging, including 2D and 3D echocardiography and fetal MRI, can be helpful in quantifying the risk of early postnatal compromise in fetal TAPVD and ensuring effective provision of postnatal resources. Familial TAPVD is well described, and careful exclusion of TAPVD by fetal echocardiography should be considered in all subsequent pregnancies once an index case is identified. Clinical genetics consultation has a role in familial cases both to establish potential causative genes and to provide appropriate genetic counseling. More objective means of interpreting prenatal imaging findings, particularly in the context of the transitional circulation, may help to improve risk stratification in the future.

## Funding Support and Author Disclosures

Dr Lloyd has acknowledged support from the British Heart Foundation (London, United Kingdom) via an Intermediate Clinical Research Fellowship (FS/ICRF/22/26028). This work was also supported by core funding from the 10.13039/501100023312Wellcome/EPSRC Centre for Medical Engineering (WT203148/Z/16/Z) and by the 10.13039/501100000272National Institute for Health and Care Research (NIHR) 10.13039/501100018835Clinical Research Facility (CRF) and HealthTech Research Center in Cardiovascular and Respiratory Medicine (HRC) at 10.13039/501100004941Guy's and St Thomas' NHS Foundation Trust, London, UK. The views expressed are those of the author(s) and not necessarily those of the NHS, the NIHR or the Department of Health and Social Care. All other authors have reported that they have no relationships relevant to the contents of this paper to disclose.
